# Characterization of polyurethane-based synthetic vertebrae for spinal cement augmentation training

**DOI:** 10.1007/s10856-018-6161-2

**Published:** 2018-09-29

**Authors:** Marianne Hollensteiner, Melanie Botzenmayer, David Fürst, Martin Winkler, Peter Augat, Sabrina Sandriesser, Falk Schrödl, Benjamin Esterer, Stefan Gabauer, Klaus Püschel, Andreas Schrempf

**Affiliations:** 1 Research Group for Surgical Simulators Linz, Upper Austria University of Applied Sciences, Linz, Austria; 2 Institute for Biomechanics, Paracelsus Medical University, Salzburg, Austria; 3 Institute of Applied Sciences and Mechatronics, Munich University of Applied Sciences, Munich, Germany; 4 Institute for Biomechanics, Trauma Clinic, Murnau, Germany; 5 Institute of Anatomy, Paracelsus Medical University, Salzburg & Nuremberg, Austria; 6 Department of Forensic Medicine, University Medical Center, Hamburg-Eppendorf, Germany

## Abstract

Vertebral augmentation techniques are used to stabilize impacted vertebrae. To minimize intraoperative risks, a solid education of surgeons is desirable. Thus, to improve education of surgeons as well as patient safety, the development of a high-fidelity simulator for the surgical training of cement augmentation techniques was initiated. The integrated synthetic vertebrae should be able to provide realistic haptics during all procedural steps. Synthetic vertebrae were developed, tested and validated with reference to human vertebrae. As a further reference, commercially available vertebrae surrogates for orthopedic testing were investigated. To validate the new synthetic vertebrae, characteristic mechanical parameters for tool insertion, balloon dilation pressure and volume were analyzed. Fluoroscopy images were taken to evaluate the bone cement distribution. Based on the measurement results, one type of synthetic vertebrae was able to reflect the characteristic parameters in comparison to human vertebrae. The different tool insertion forces (19.7 ± 4.1, 13.1 ± 0.9 N, 1.5 ± 0.2 N) of the human reference were reflected by one bone surrogate (11.9 ± 9.8, 24.3 ± 3.9 N, 2.4 ± 1.0 N, respectively). The balloon dilation pressure (13.0 ± 2.4 bar), volume (2.3 ± 1.5 ml) of the synthetic vertebrae were in good accordance with the human reference (10.7 ± 3.4 bar, 3.1 ± 1.1 ml). Cement application forces were also in good accordance whereas the cement distribution couldn’t be reproduced accurately. Synthetic vertebrae were developed that delivered authentic haptics during transpedicular instrument insertion, balloon tamp dilation and bone cement application. The validated vertebra model will be used within a hybrid simulator for minimally invasive spine surgery to educate and train surgeons.

## Highlights

- Anatomically realistic open-celled vertebrae based on polyurethane and calcium-based mineral fillers were developed.
- Spine surgery specific testing of human and synthetic vertebrae were conducted.
- The examined tool insertion forces and balloon tamp dilation pressures of artificial vertebrae were highly comparable to human ones.
- Open-celled artificial vertebrae enabled a realistic bone cement distribution.
- Realistic haptic performance and behavior of two composite materials confirmed their suitability for training in the field of spine surgery.

## Introduction

Elderly people often suffer from a fracture of an osteoporotic vertebral body, when they complain about suddenly occurring non-traumatic back pain. In 2010 about 27 million Europeans were affected by vertebral compression fractures (VCF) caused by osteoporosis [1], which mostly affect the thoracolumbar region [2]. If pain-problems after a first line conservative, non-surgical therapy persist, surgical intervention may be considered. Vertebral augmentation techniques, namely vertebroplasty and kyphoplasty, are the treatments of choice. Such percutaneous techniques stabilize an affected vertebra from within by creating a fluoroscopy-guided bone access to the vertebral body followed by an injection of bone cement for the stabilization of bone fragments. The main difference between vertebro- and kyphoplasty is, that the latter additionally uses an inflatable balloon tamp in order to restore the vertebral height and to provide improved spinal alignment [3]. The transpedicular approach represents the classic approach for most augmentation techniques since the pedicle offers an anatomical landmark for needle targeting and minimize the risk of damage for surrounding structures. However, it is considered only a safe technique as long as an intrapedicular pathway is maintained [4] and an inaccurate needle placement may lead to injuries of the nerve root, the spinal cord or even adjacent organs, e.g. the lungs [5]. Another complication for both techniques is cement leakage. Since the vertebral body is highly vascular, there is a risk of extravasation of the bone cement into the circulatory system. This may lead to undesired systemic effects like hypotension, respiratory distress and pulmonary or cerebral cement embolism. Further, a cement leakage into the spinal canal is reported resulting in pain exacerbation [4], paraplegia or radiculopathies [6]. In a review by Garfin et al. cement leakages were reported in 34 to 64% of all vertebral augmentation treatments [3]. To prevent symptomatic cement leaks the use of a high-resolution fluoroscopy and barium sulfate-enriched bone cements providing an adequate level of opacification are recommended allowing an interruption of the cement injection when a leak is recognized. Risks associated with kyphoplasty are the breakage of a pedicle since typically bigger needles are used in this maneuver to enable placement of the balloon tamp in the vertebral body [6]. Further, the rupture of the balloon tamp is reported in one out of three kyphoplasty treatments. However, this is only the case after full inflation [7].

Typical pathways to teach surgical skills follow the Halstedian approach of “learning by doing”, studies on human or animal specimens, (computer-based) models or human patient simulators. Nowadays, the surgical education and the acquisition of surgical skills are trained in traditional clinical or simulated environments [8]. Several studies demonstrated, that haptic feedback during surgical training enhances learning [9–12] and furthermore, that surgical skills are significantly increased with the help of realistic tactile feedback compared to visual learning alone [13, 14]. With the help of surgical simulators, novice surgeons are allowed to train surgical procedures without limiting factors like the immense costs of specimens, the exposure of radiation due to fluoroscopic guidance or the requirement of a mentor [15]. To improve education of surgeons as well as patient safety, we recently initiated the development of a high-fidelity simulator for the surgical training of cement augmentation techniques [16]. The simulator consists of a realistic patient phantom and a fluoroscopy simulation. The patient-phantom composed of validated synthetic vertebrae [17] and soft tissues [18], allows a realistic transpedicular bone access. The synthetic vertebrae consist of an outer rigid shell mimicking the cortical layer and closed-cell polyurethane foam imitating the trabecular structure [17]. Original rigid bone access tools, which are used within the simulator, are equipped with sensor coils providing the position and orientation of the tools. This data allows the visualization for simulated fluoroscopy projections as well as a 3D representation of tools and vertebrae [16]. Due to the fact that the trabecular structure of the synthetic vertebrae was imitated by a closed-cell polyurethane foam, realistic dilation of a balloon tamp or the application of injected bone cement was not possible yet [19]. Therefore, the aim of this project was the characterization of new synthetic vertebrae with an open-celled trabecular structure to enable training of the whole surgical procedure including transpedicular tool insertion, balloon tamp dilation and bone cement injection. We were specifically interested in the characterization of haptic parameters of cement augmentation techniques and to use these parameters to validate new synthetic vertebrae with an open-celled trabecular bone against human vertebrae.

## Materials and methods

### Human specimen

Six fresh frozen human thoracolumbar spines (Th9 to L5) of elderly women (76.2 ± 9.4a, 161 ± 3 cm, 59 ± 6 kg, BMD 92.1 ± 35.0 mg/cm^3^) were thawed, the vertebrae were dissected from the spines and soft tissues were removed. Vertebrae with visible defects of the cortical bone layer were excluded. The remaining 38 segments were equally divided into two groups. Group VERTEBRO vertebrae (n = 20) were treated with cement application according to the surgical procedure of vertebroplasty (transpedicular needle insertion and cement application) [3] and the vertebrae of group KYPHO (n = 18) were treated with balloon-kyphoplasty (transpedicular needle insertion and balloon tamp dilatation) [7]. Human material was obtained through the anatomical gifts program at the University Hamburg-Eppendorf, Germany, in accordance with the Human Tissue Act [20] and were approved by the ethics committee of the state Bavaria, Germany.

### Artificial vertebrae

Three different groups of synthetic vertebrae (SV), level L3 to L5, mainly consisting of polyurethane (PU) resin were molded in a two-step process. First, a predetermined amount of a “cortical” PU mixture was casted in a silicone-based negative mold of a human vertebra for approximately 3 min. Due to a continuous rotation of the mold in a rotating casting machine, the PU mixture accumulated to the negative mold thus creating a hollow vertebra. Another “cancellous” mixture was injected into the hollow vertebra with a syringe to create a cancellous padding. The composition of the different groups of synthetic vertebrae, a detailed description of the materials and the molding process is described elsewhere [21]: the synthetic cancellous bone of SV1 and SV2 were enriched with calcium-phosphate whereas SV3 was filled with polyurethane edited with calcium-carbonate-powder. Additionally, varying amounts of blowing agents were used to create an open-celled structure (0.75%_*wt*_ for SV1, 1.0%_*wt*_ for SV2 and 1.25%_*wt*_ for SV3). As a further reference, commercially available vertebrae (SB, *n* = 5, model No. 1378–60–4, Sawbones Europe AB, Malmö, Sweden) made of a cancellous foam and a rigid cortical shell, both based on polyurethane, were obtained. This type of vertebrae was selected because it is the most commonly used for surgical training.

### Transpedicular needle insertion

**Fig. 1:**
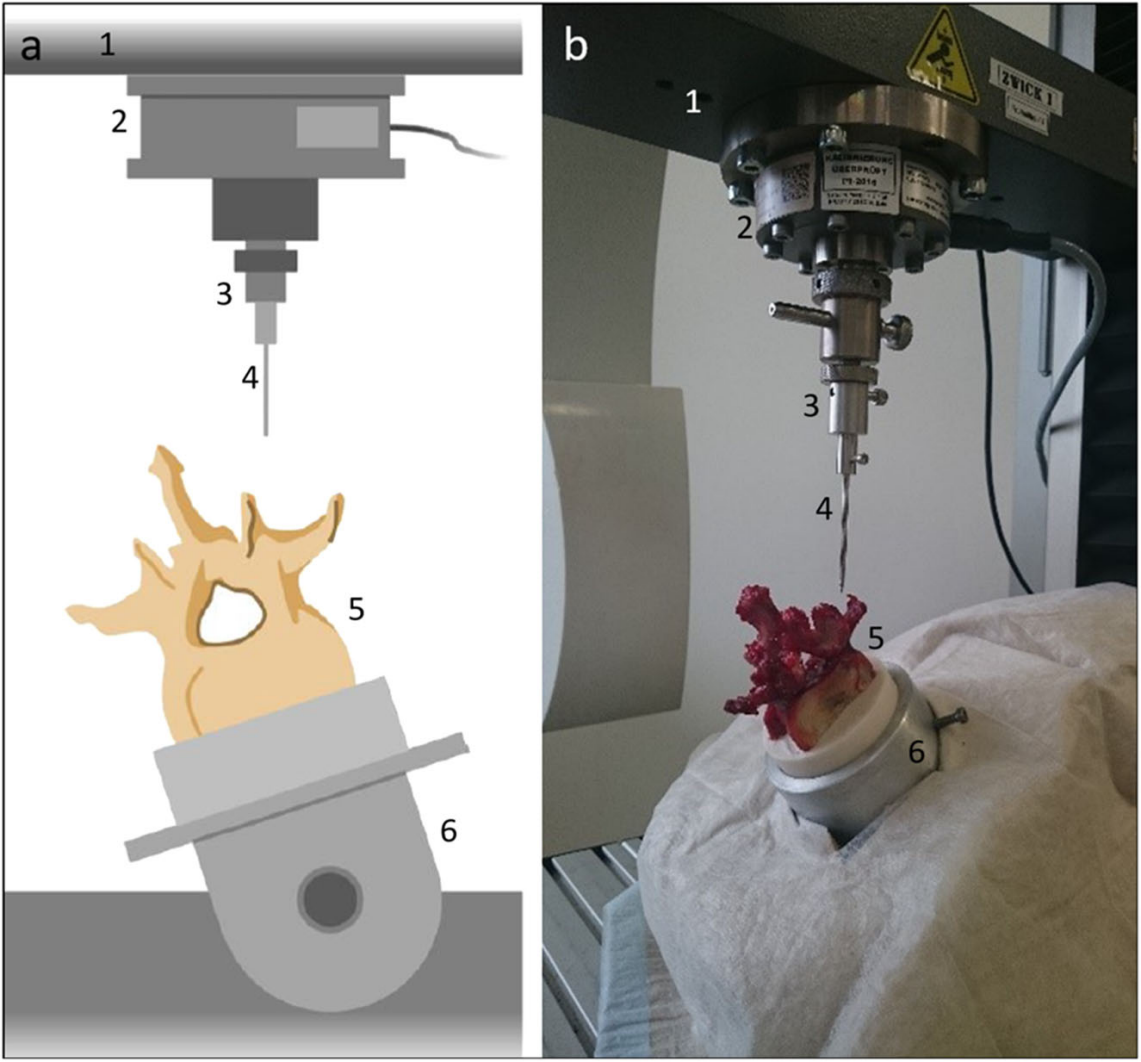
Schematic view **a** and real measurement setup **b** of transpedicular needle insertion. (1: material testing machine, 2: load cell, 3: adapter for Jamshidi needle, 4: Jamshidi needle, 5: specimen, 6: 3D bench vise with embedding pot)

Transpedicular instrument insertions were performed as described in a previous study [17]. In short, the vertebrae were embedded in an embedding pot with a fast curing resin (Rencast FC53 A/B, Huntsman Advanced Materials, Basel, Switzerland). The tip of an 11 G diamond Jamshidi needle (Kyphon Inc., KyphX Osteo Introducer, Sunnyvale, CA) was centered and fixed on the load cell in an uniaxial testing machine (Zwick Z010, Zwick Roell, Ulm, Germany). The pedicle of the vertebrae was aligned with the axis of the Jamshidi needle to enable an automatic transpedicular access of the needle into the vertebral body (see Fig. 1). The Jamshidi needle was driven into the pedicle with a feed rate of 1 mm/s until an insertion depth of 30 mm was reached. The resulting load- displacement data were recorded.

A parametric model for the calculation of needle insertion forces [21] was used to identify the characteristic instrument insertion parameters. The needle insertion forces were divided into a cortical (*F*_*cort*_ (*x*)) and a cancellous force (*F*_*canc*_ (*x*)) which depend on the needle’s insertion depth x. *F*_*cort*_ (*x*) reflects the initial puncturing of the cortical layer of the vertebrae and was modeled by a radial bias function with two centers and two spreads. Based on the needle insertion measurement data, two weighting factors *ω*_1_ and *ω*_2_ were calculated. Their average (further denoted as (*ω*_1_, *ω*_2_)) was used as the first characteristic parameter for the evaluation of transpedicular needle insertion. The cancellous force can be further divided into a constant cutting force (*F*_*cut*_ (*x*)) and a linear increasing clamping force (*F*_*clamp*_ (*x*)). As a second characteristic parameter, the cutting force *F*_*cut*_ (*x*) was modeled, which depends on structure and density of the trabecular bone. This cutting force appeared when the whole needle tip penetrated through the cortical layer. It’s mean value was constant. Further, a clamping force, which represented the friction between bone and instrument, was modeled with the help of a unit step function (Heaviside function). *f*_*clamp*_ (*x*), which denotes the clamping force per insertion depth, was used as a third characteristic parameter. Due to bi- and transpedicular instrument insertion measurements performed with all specimens, the sample number of needle insertions doubled.

### Kyphoplasty balloon dilation

**Fig. 2:**
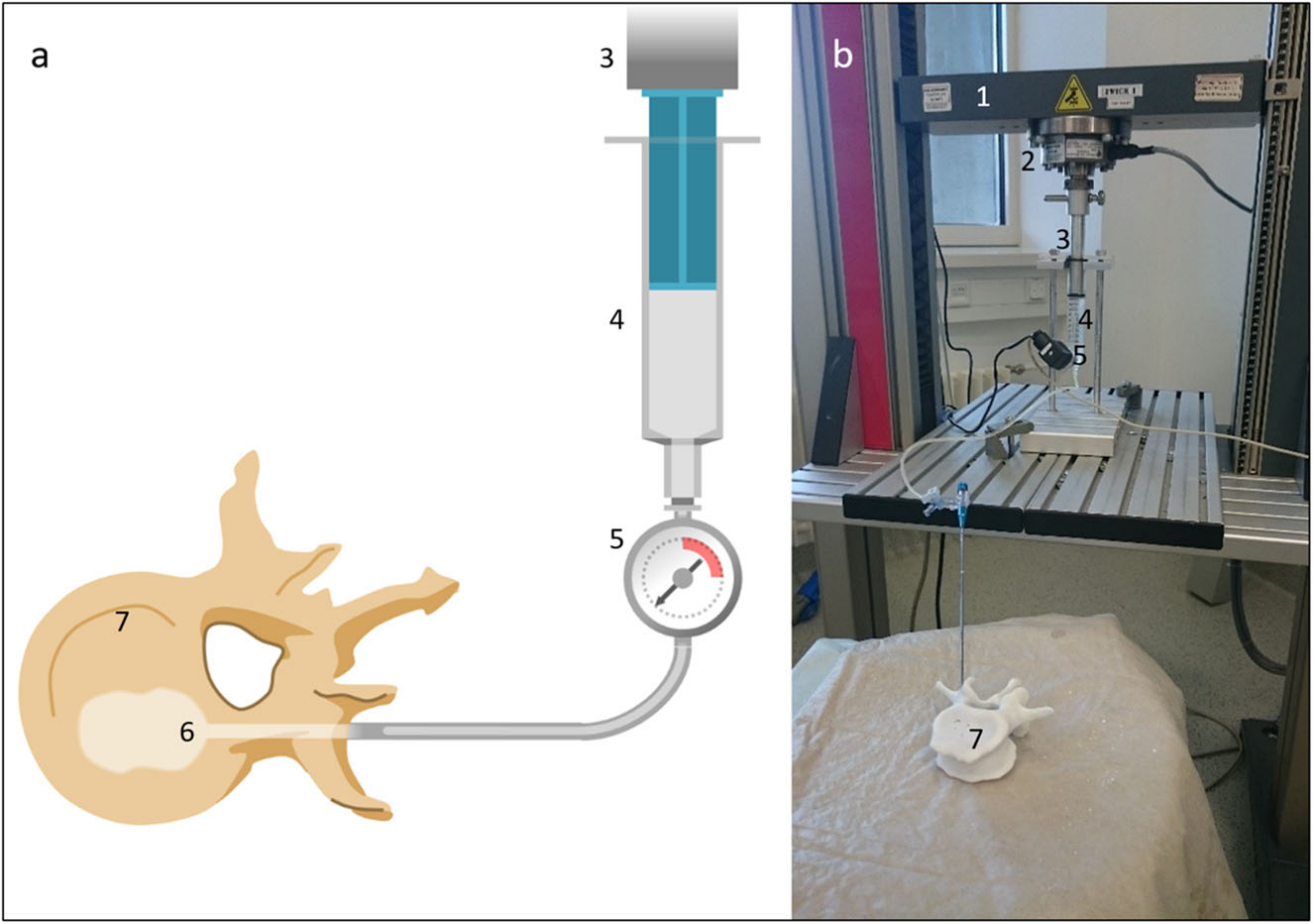
Schematic view **a** and real measurement setup **b** of kyphoplasty balloon dilation measurements (1: material testing machine, 2: load cell, 3: plunger, 4: ballon expander, 5: pressure sensor, 6: balloon tamp, 7: specimen)

The needle insertion canal of group KYPHO vertebrae, which was previously created by the insertion of the 11 G Jamshidi needle, was widened by a manual insertion of an 8 G Jamshidi needle. Then, the inner trocar of the 8 G needle was removed and a bone drill was inserted through the remaining cannula. A cavity of 20 mm length was manually drilled in the trabecular bone of the vertebral body, overall creating a canal of 50 mm length. After the removal of the drill, a 20 mm balloon tamp (uDuro, ulrich medical, Ulm, Germany) was introduced in the previously created insertion canal (see Fig. 2). Further, a commercial kyphoplasty balloon dilation pump (uDuro, ulrich medical, Ulm, Germany) was adapted for measurement reasons. The plastic plunger of the pump was replaced by a custom made plunger made of aluminum in order to avoid elastic deformation. The plunger was sealed with a sealing ring and was vertically mounted to the load cell of the material testing machine. The housing of the pump was vertically fixed in the material testing machine and was aligned to the plunger. The analogue pressure sensor of the dilation pump was replaced by a digital sensor (A-10, accuracy 0.5%, Wika GmbH & Co KG, Trennfurt am Main, Germany) to acquire a higher accuracy. Afterwards, the pump was filled with radiopaque contrast agent (Ultravist300, Bayer Vital GmbH, Leverkusen, Germany) to enable fluoroscopy visualization. For balloon tamp dilation measurements, the plunger was driven with a feed rate of 1 mm/s until a force of 50*N* was reached. Then the measurement was stopped and a fluoroscopy image was taken to control the placement and dilation of the balloon tamp. This process was repeated and the forces were increased in steps of 50*N* until the expanding balloon touched the cortical layer of the vertebral endplates or the balloon was sufficiently dilated according to the opinion of an experienced surgeon. Measurements exceeding an application pressure of 25 *bar* were discarded. The applied pressures and volumes during the balloon dilations were recorded.

### Vertebroplasty bone cement application

**Fig. 3:**
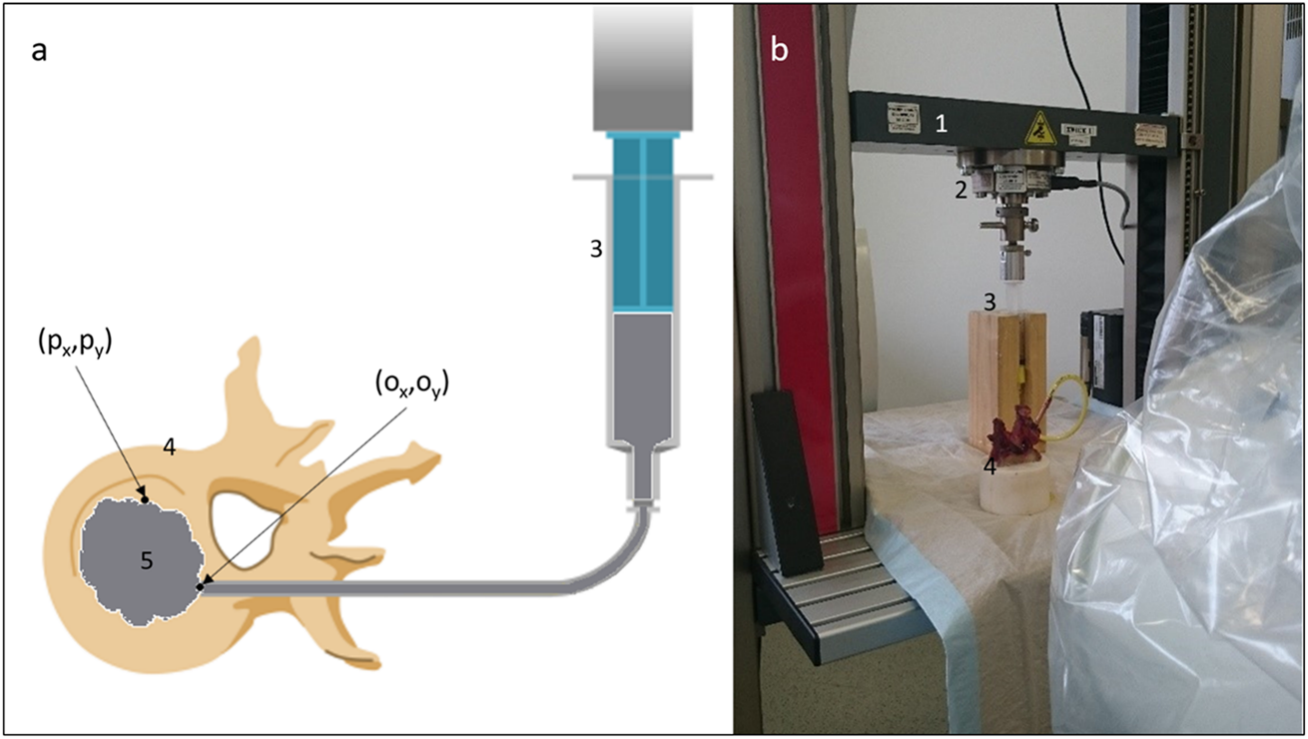
Schematic view (**a**) and real measurement setup (**b**) of vertebroplasty cement application measurements (1: material testing machine, 2: load cell, 3: syringe filled with cement, 4: specimen, 5: bone cement)

Again, needle insertion measurements were performed and the transpedicular access was widened with an 8 G working cannula. Then, the inner trocar was removed and a tube with a diameter of 4 mm equipped with a Luer Lock adapter was attached to the remaining cannula. A synthetic bone cement for training purposes [22] was prepared and 15 ml were distributed into a syringe. The tube was locked to the syringe with the Luer Lock adapter. The syringe was fixed in a custom made housing and was placed upside down in the universal testing machine (see Fig. 3). The plunger of the syringe was pushed with a constant feed rate to deliver 1 ml of cement into the vertebra with a cement flow rate of 0.15 ml/s [23]. Then the measurement was stopped and a fluoroscopy image was taken to control the distribution of the bone cement. This process was repeated until 6 ml were applied into the vertebra, the cement was sufficiently distributed according to the opinion of an experienced surgeon or cement leakage occurred. After each step, a fluoroscopy image was taken in transverse plane [23].

To compare the flow behavior of the cement in human and synthetic vertebrae, the synthetic bone cement was prepared according to Hollensteiner et al. [22] and identical starting times were adhered. The application force (*F*_*app*_) and applied cement volume were recorded. Further, the distribution of the cement in the cancellous bone was assessed according to Löffel et al. [23]: the fluoroscopy images were processed with Matlab (Matlab2016a, The Mathworks, Nattick, USA). The cement was automatically detected and the circularity (CIRC) of the cement spread and the mean cement spreading distance (MCSD) were assessed. Therefore, the fluoroscopy image of the untreated vertebra was subtracted from the image which shows the cemented vertebrae to create an image of the remaining bone cement. The images were filtered by a median filter to reduce noise and were converted into binary images. Further, the boundaries of the cement were detected. The circularity was assumed as a measure for compactness by comparing the area (*A*_*s*_) and the perimeter (*P*_*s*_) of the spread cement shape (*S*) compared to circle (*P*_*c*_) with the same perimeter [23] (see Eq. (1)):

1 $$CIRC = \frac{{P_c}}{{P_s}} = \frac{{2 \ast \sqrt {\pi \ast A_s} }}{{P_s}} \ast 100$$

Increasingly elongated or bulky contours approach 0 and a perfect circular shape has a CIRC of 1. However, the CIRC parameter was invariant of the size of the circle. To investigate the size, the mean cement spreading distance (MCSD) was calculated. Therefore, the Euclidian distance from the fluoroscopy image pixels filled with cement (*P*_*i*_ = (*p*_*x*_, *p*_*y*_)) to the origin of the cement application (*O* = (*o*_*x*_, *o*_*y*_)) was computed [23] (see Eq. (2)):

2 $$\begin{array}{*{20}{l}} {{\mathrm{MCSD}}} \hfill & = \hfill & {\frac{1}{n}\mathop {\sum}\limits_{i = 1}^n \sqrt {\left( {p_x - o_x} \right)^2 + \left( {p_y - o_y} \right)^2} } \hfill \\ {} \hfill & {} \hfill & {(\forall \,P_i\,filled\,with\,cement)} \hfill \end{array}$$

### Statistical analysis

Data were tested for normal distribution with Shapiro–Wilk test. Normally distributed data were further assessed for homogenous variances with Levene test. Normally distributed and homogenously varied data were tested with unpaired Student’s t-test to detect differences between the human and synthetic sample groups. Otherwise, Mann–Whitney-U test was used. For all tests, a p-value of 0.05 or less was considered significant.

## Results

### Needle insertion

**Fig. 4:**
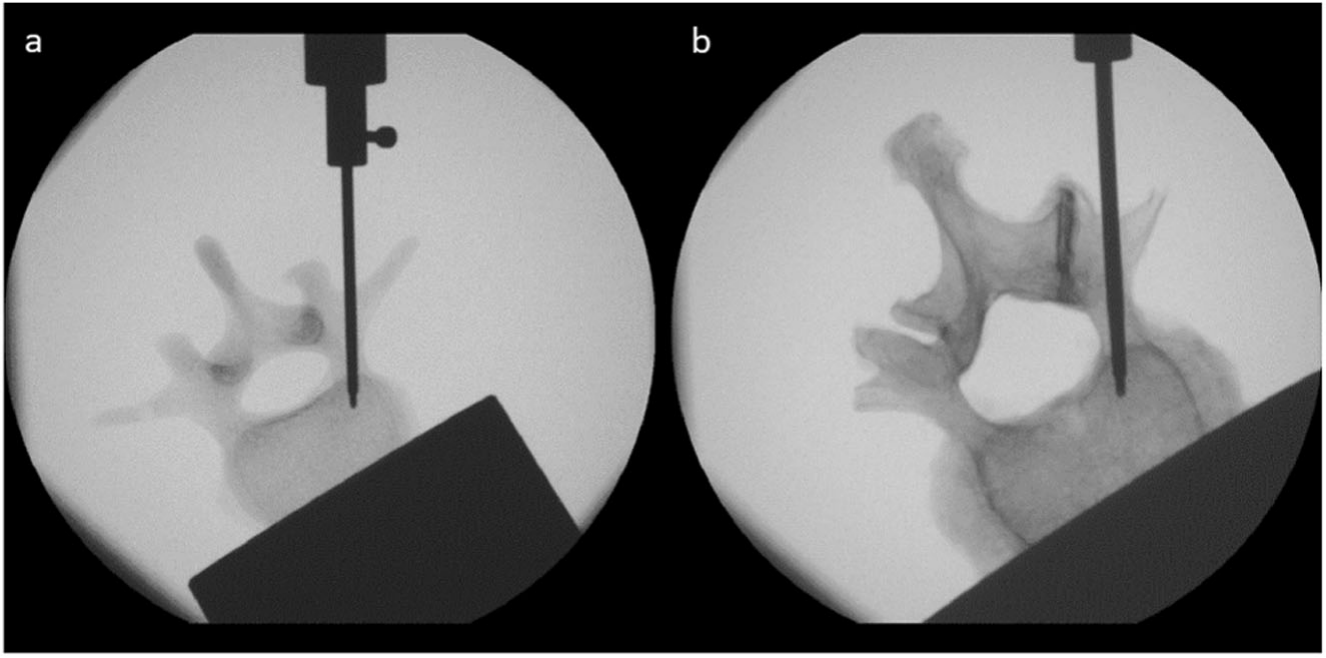
Fluoroscopy image during transpedicular needle insertions (**a** synthetic vertebra; **b** human vertebra)

The results of the transpedicular needle insertion measurements are summarized in Table 1. Prior to evaluation of the needle insertion data, the fluoroscopy images (Fig. 4) were analyzed. Measurements with incorrect needle placements or measurements including a deflection of the insertion tool, which would result in deflecting forces, were eliminated from the study. The average weighting factors were lowest for the SV2 sample group followed by the commercially available SB and the SV3 sample group. The mean weighting factors of SV2 and SB were approximately 20% smaller than those obtained from the human vertebrae. The highest weights were detected for SV1, whose parameters were similar to the results obtained from the human reference. Statistically significant differences to the human vertebrae were only detected for SV3 (*p* = 0.006). The cutting forces obtained from SV3 and SB showed significant differences to the results of the human samples (*p* = 0.021 and *p* = 0.001, respectively); the mean parameter of SB was more than 3 times larger than the forces measured from the human bones. The customized synthetic vertebrae SV2 was in good accordance with the human vertebrae (*p* = 0.220). The clamping force was lowest for SV3, followed by SB, which were 52 and 48% smaller than the modeled clamping forces of the human vertebrae. The mean clamping forces of SV1 and SV2 were 32 and 10% larger than the human sample group. However, statistically significant differences were only detected for SV3 (*p* < 0.001). The samples SV1 (*p* = 0.354), SV2 (*p* = 0.495) and SB (*p* = 0.343) did not show significant differences.

**Table 1:** Results of needle insertion measurements in fresh frozen human vertebrae (Human), three varying customized bone surrogates (SV1, SV2, SV3) and a commercially available bone model (SB) (mean ± standard deviation (p-value in comparison to human bones))

	*Human (Group VERTEBRO, n* = 34*)*	*SV1 (n* = 10*)*	*SV2 (n* = 62*)*	*SV3 (n* = 49*)*	*SB (n* = 8*)*
*ω*_1_, *ω*_2_	32.7 ± 20.3	35.2 ± 32.9 (0.989)	24.8 ± 17.6 (0.051)	28.2 ± 24.9 (0.006^*^)	26.9 ± 10.0 (0.731)
*F*_*cut*_ [N]	24.4 ± 20.4	40.2 ± 27.6 (0.136)	28.1 ± 19.8 (0.220)	17.9 ± 17.5 (0.021^*^)	78.0 ± 24.9 (0.001^*^)
*f*_*clamp*_ [N/mm]	2.8 ± 2.0	3.8 ± 2.7 (0.354)	3.1 ± 1.9 (0.495)	1.4 ± 1.3 (<0.001^*^)	1.5 ± 0.0 (0.343)

^∗^p < 0.05

### Balloon dilation

**Fig. 5:**
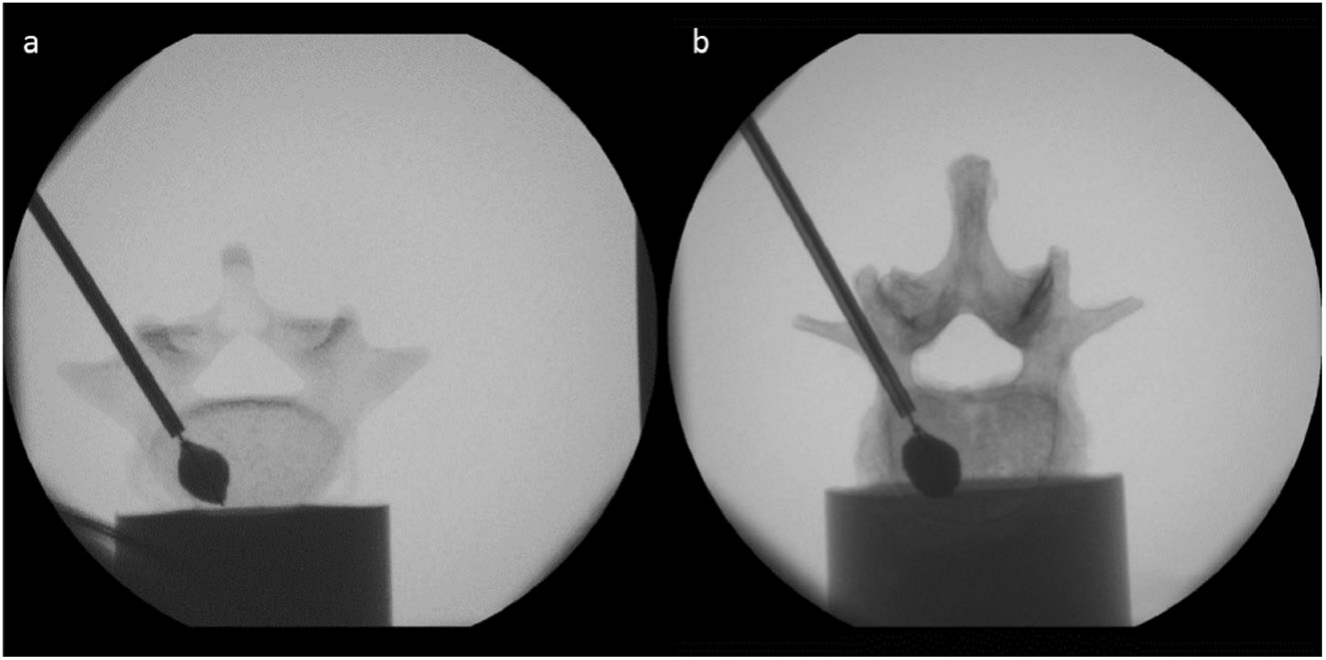
Fluoroscopy image during balloon dilation in an synthetic (**a**) and a human (**b**) vertebra

The results of the kyphoplasty balloon dilation measurements are summarized in Table 2. Before evaluation of the balloon dilation data, the fluoroscopy images (see Fig. 5) were analyzed. Mesasurements with undilated balloon tamps, tamps with an early contact with a cortical endplate or breakage of the balloon through the cortical wall were eliminated from the study. Balloon dilation measurements of the SV1 and SB weren’t possible. The balloon couldn’t be inflated within the rigid trabecular structure leading to excessively high pressures over 25 bar. Thus, the SV1 samples were excluded from the statistical evaluation.

**Table 2:** Results of kyphoplasty balloon dilation measurements in fresh frozen human vertebrae (Human), two varying customized bone surrogates (SV2, SV3) (mean ± standard deviation (p-value in comparison to human bones))

	*Human (Group KYPHO, n* = 19*)*	*SV2 (n* = 20*)*	*SV3 (n* = 16*)*
*V*_*max*_ [ml]	3.1 ± 1.1	4.4 ± 1.4 (0.472)	2.3 ± 1.0 (0.101)
*P*_*max*_ [bar]	10.7 ± 3.4	12.3 ± 2.1 (0.082)	12.3 ± 2.7 (0.042^*^)

^∗^p < 0.05

The balloon volumes of the synthetic vertebrae SV2 and SV3, which were approximately 40% larger and 26% smaller than the volumes measured in the human vertebrae, were comparable to volumes obtained from the human sample group (*p* = 0.472 and *p* = 0.101, respectively). The maximum applied pressure (*P*_*max*_) for the balloon dilation was highest for SV2, followed by the SV3 and the human sample group. Both synthetic sample groups were about 15% larger than the human sample group. SV3 was statistically different to the human reference (*p* = 0.042) whereas SV2 showed comparable balloon pressure values (*p* = 0.082).

### Bone cement application

**Fig. 6:**
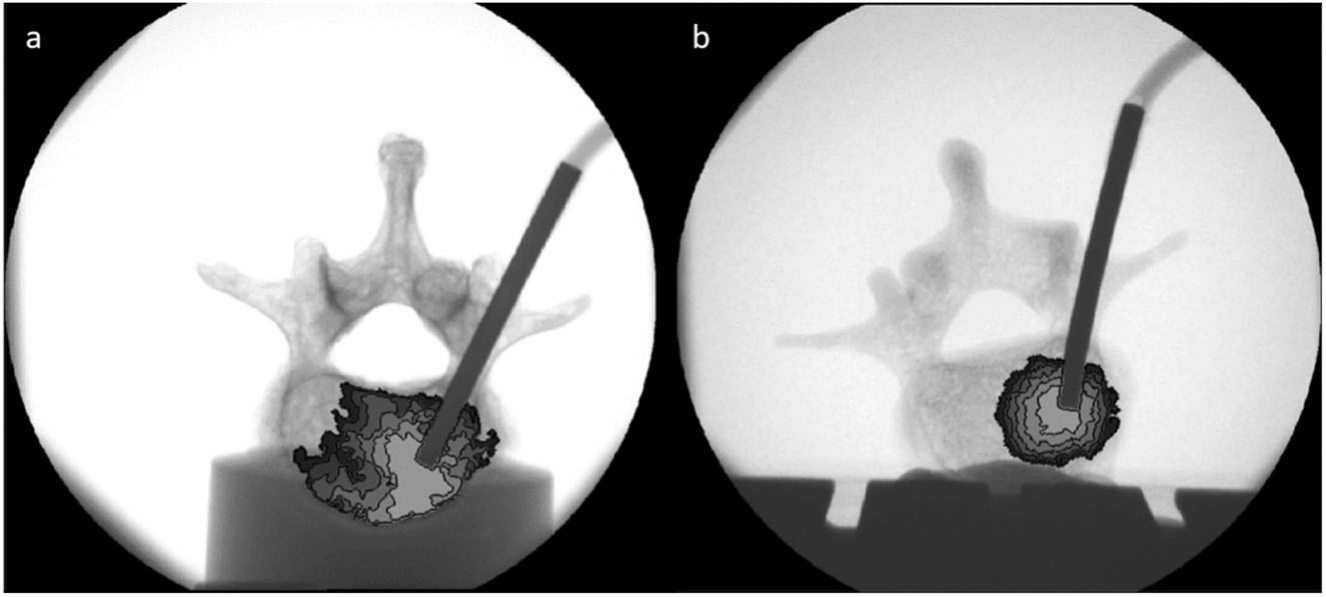
Adapted fluoroscopy images of cement applications (**a** human bone, **b** synthetic bone). After cement application, the distributed cement was colored in ascending colors (from 1 ml (light grey) to 6 ml (black))

The results of vertebroplasty cement application measurements are summarized in Table 3. Before evaluation of cement distribution, the fluoroscopy images (see Fig. 6) were analyzed to exclude measurements with cement leakage. Furthermore, the distribution of the cement was presented in ascending colors within the fluoroscopy images leading to a color map of the cement distribution (see Fig. 6). Cement applications were not possible in SV1 and SB vertebrae. Distribution of cement could not be detected within their cancellous structure, but an extreme rise of application forces (>120 N) and a leakage of the system were detected. Thus, these two sample groups were excluded from the statistical analysis. The mean cement spread distance (MCSD) was calculated after each distribution of 1 ml of cement for six cycles, resulting in a final cement application volume of 6 ml. Overall, the MCSDs of the synthetic vertebrae were smaller during all cement application steps than the MCSDs of the human vertebrae. On average, the MCSD of SV3 was 35% smaller and the MCSD of SV2 was 45% smaller compared to the MCSD of the human vertebrae. Statistical differences were detected for all application steps for the SV2 and for the last four milliliters of cement which were applied into SV3. No differences were detected for 1 and 2 ml applications in SV3 compared to the same application amounts in the human vertebrae (*p* = 0.094 and *p* = 0.092). The circularity (CIRC) percentage of the distributed cement of all application steps and all synthetic vertebrae was significantly higher than the results obtained from human vertebrae. Compared to the circularity of human vertebrae, the CIRC of SV2 were clearly larger and ranged from 38% (1 ml) to approximately 49% (5 ml). Similarly, the CIRC of SV3 was also larger compared to the CIRC of the human vertebrae and exceeded the human CIRC values from 33 to 58%. The measured cement application forces (*F*_*app*_) of SV2 and SV3 vertebrae did not show significant differences compared to the application forces gathered from the cement application in human vertebrae, except for the 5 and 6 ml application measurements of the SV2. On average, *F*_*app*_ of SV2 was only 15% larger than Fapp of the cement application in human vertebrae while *F*_*app*_ was in good accordance for SV3 with average deviations of only 2%.

**Table 3:** Results of cement application in fresh frozen human vertebrae (Human) and two varying customized bone surrogates (SV2, SV3) (mean ± standard deviation (p-value in comparison to human bone))

	*Cement Vol. [ml]*	*Human Group KYPHO (n* = 15*)*	*SV2 (n* = 15*)*	*SV3 (n* = 15*)*
*MCSD* [mm]	1	15.2 ± 6.8	8.1 ± 4.1 (0.006^*^)	9.9 ± 2.7 (0.094)
	2	15.9 ± 5.8	8.8 ± 2.7 (0.003^*^)	10.8 ± 1.9 (0.092)
	3	17.0 ± 4.58	9.4 ± 2.7 (<0.001^*^)	11.5 ± 1.4 (0.003^*^)
	4	18.4 ± 4.2	10.2 ± 2.9 (<0.001^*^)	12.3 ± 1.5 (0.001^*^)
	5	19.6 ± 5.0	10.9 ± 2.9 (<0.001^*^)	12.7 ± 1.0 (0.001^*^)
	6	20.6 ± 4.3	11.6 ± 3.2 (<0.001^*^)	13.2 ± 1.7 (0.001^*^)
*CIRC* [%]	1	48.9 ± 12.3	67.7 ± 15.5 (0.006^*^)	65.2 ± 9.1 (0.013^*^)
	2	41.8 ± 10.7	62.6 ± 13.6 (0.002^*^)	64.8 ± 5.6 (0.001^*^)
	3	39.7 ± 7.0	54.6 ± 10.0 (0.006^*^)	59.4 ± 5.9 (0.001^*^)
	4	39.3 ± 8.5	57.1 ± 7.4 (<0.001*)	60.3 ± 7.1 (0.001*)
	5	38.2 ± 8.6	56.7 ± 8.9 (0.001^*^)	60.3 ± 7.6 (0.001^*^)
	6	40.1 ± 10.9	56.7 ± 6.4 (0.001^*^)	58.4 ± 7.1 (0.003^*^)
*F*_*app*_ [*N*]	1	45.2 ± 10.3	44.4 ± 5.5 (0.689)	48.2 ± 7.9 (0.123)
	2	41.6 ± 5.5	47.2 ± 7.4 (0.114)	47.1 ± 11.2 (0.180)
	3	43.0 ± 8.4	47.1 ± 3.6 (0.267)	44.7 ± 11.9 (0.689)
	4	47.0 ± 9.9	54.9 ± 4.2 (0.114)	49.2 ± 15.9 (1.000)
	5	53.5 ± 12.0	66.2 ± 6.8 (0.032^*^)	51.3 ± 19.7 (0.424)
	6	59.2 ± 13.3	76.0 ± 8.8 (0.017^*^)	57.0 ± 19.4 (0.465)

^∗^p < 0.05

## Discussion

This study was focused on the procedural characterization of custom-made synthetic vertebrae which can be used in a hybrid simulator for the training of vertebroplasty and kyphoplasty. Automated measurements of transpedicular tool insertion force, balloon tamp dilation pressure and volume and bone cement application forces and its distribution were performed and compared to human specimens.

Although the synthetic vertebrae were all manufactured with the same material mixtures as described in the pre-study by Hollensteiner et al. [21], the synthetic vertebrae achieved better results for transpedicular tool insertion than the pre-study blocks. In the pre-study, nearly all material blocks showed significant differences for the investigated cutting parameter *F*_*cut*_ and the clamping force *f*_*clamp*_. This can be explained by the molding process of the vertebrae which led to non-uniform cortical layers ranging from 0.2 to 1.3 mm. The cortical layer of human vertebrae ranges from 1.3 ± 1.2 mm [24], thus the non-uniform cortical thickness of the synthetic vertebrae is more realistic than the uniform 1.5 mm-layer of the pre-study blocks. Further, the expansion of the trabecular PU foam was not that straightforward due to the tight and narrow processes and pedicles of the closed vertebra molds. Therefore, varying cancellous foam densities were obtained during the molding process, which influenced the clamping forces. While the obtained variances in bone structures are unacceptable for studies comparing biomechanical properties of bones or implants, these variances are entirely desirable for surgical training and education to create varying training scenarios [25]. In contrast, the cancellous structures within the pedicles of the SB vertebrae were directly proceeding to the inferior articular process, leading to a very thick cortical layer between the inferior articular process and the transverse process of the synthetic vertebrae. However, according to Mobbs et al. [26] transpedicular tools are inserted more laterally between the two aforementioned structures of the inferior articular process and the transverse process. The cortical layer of the synthetic vertebrae from Sawbones ranges from 3 to 5 mm whereas the cortical thickness of the custom made vertebrae ranged from 0.2 and 1.2 mm. As mentioned above, the cortical layers at the transpedicular tool insertion point at human pedicles are 1.3 ± 1.2 mm [27]. Thus, the cortical thickness of the custom vertebrae was more realistic. The tool insertion measurements demonstrated that the synthetic vertebrae SV1 and SV2 had a similar mechanical behavior during needle insertion compared to the human reference while SV3 and SB demonstrated considerable differences. The synthetic trabecular bone of SV1 and SV2 was filled with calcium phosphate whereas SV3 was made with calcium carbonate. The latter has a higher degree of material density leading to stiffer and more brittle synthetic trabecular bone. The cortial cutting and clamping forces of SV3 were significantly higher than the results of the human reference although a higher amount of blowing agent was used for SV3 compared to SV1 and SV2. The more blowing agent was utilized, the smaller were the clamping forces of the custom made synthetic vertebrae. The same effect was observed for the cutting forces. The measurement results showed a decline of forces with an increase of the amount of blowing agent. Further, the custom made synthetic vertebrae were all molded with an identical cortical layer. However, large variations in the calculated weighting factors, which are a parameter for the cortical tool insertion, were observed. This is due to the fact, that after penetration of the cortical layer, the chance to hit underlying trabeculae was more likely for SV1 than for SV2 because of the smaller amount of blowing agent used. Thus, a smaller amount of gas emerged and smaller bubbles but larger trabeculae were created. Although the blowing amount of SV3 was the largest one, the underlying trabeculae were created of the stiffer calcium carbonate, which led to an increase of the cortical insertion forces. The measured maximum dilatation pressure and volumes were consistent with the study from Cui et al. [28]. These authors measured the balloon peak pressure and balloon volume within a retrospective analysis of 93 patients with 154 vertebrae of osteoporotic compression fractures who received percutaneous balloon kyphoplasty. They further demonstrated that kyphoplasty balloon dilation volume in human vertebrae ranges from 2.1 to 6.3 ml and the balloon dilation pressure ranged from 130 to 359 psi which is approximately 9 to 25 bar. The results of the balloon dilation measurements demonstrated, that the mean maximum volume of the human and certain synthetic vertebrae were in good accordance with the results obtained by Cui et al. While the balloon dilation wasn’t possible in SV1 and SB vertebrae, the results of SV2 and SV3 were in good accordance with the results of the human reference and with the results obtained by Cui et al. [28]. The measured cement application parameters, mean cement spread distance (MCSD) and circularity (CIRC), were initially investigated by Löffel et al. [23] in synthetic structures and human vertebrae. They showed, that the cement viscosity, the porosity of the vertebrae and the flow rate influenced the cement spreading distance and it’s circularity. An amount of 4 ml of bone cement was injected in four human vertebrae. These results were in good accordance with the results obtained in this study where MCSDs of 17.11 ± 2.61 mm and CIRCs of 0.38 ± 0.08 were calculated. However, in our measurements a slightly higher flow rate of 0.15 ml/s and a viscosity of approximately 50 Pas were used. The color maps of the cement applications into the synthetic vertebrae appeared like annual rings of a tree whereas the color maps of the human vertebrae looked more abstract like the elevation profile of a geographical map. The distributed cement in the synthetic vertebrae showed higher circularity percentages which indicated that the cement distribution was more round-shaped. Concurrently, the mean cement spread distance was smaller, compared to the human vertebrae and hardly any outgrowths were visible. In contrast, the cement in the human vertebrae was less roundly shaped while lower circularities and larger mean cement spread distances occurred within the human vertebrae which can be explained by the characteristics of the venous system of vertebrae. The venous system of a vertebra is lacking valves and can be divided into three intercommunicating parts. The veins of the venous plexus surround the dura mater within the spinal canal, the basivertebral veins run horizontally through the vertebral body and the external vertebral venous plexus surrounds the vertebral column [29]. However, the applied cement displaced the bone marrow fat and other liquids in the trabecular spaces. Additionally, the cement entered the basivertebral veins following the way of less resistance. Furthermore, in some rejected measurements, a cement leakage into the spinal canal was visible. In one extreme case, the whole distributed cement only gathered into the venous plexus and thus, the spinal canal, and hardly any cement was visible within the trabecular bone. Thus, the correct placement of the cannula in the cancellous bone is mandatory in order to avoid cement leakage. Due to the manufacturing process, where the PU foam uniformly expands in the cortical shell, the venous system cannot be built. Thus, no leakage in the spinal canal nor other surrounding soft tissue or vascular parts would be possible. The circularity of the two custom made vertebrae was approximately the same, although a higher amount of blowing agent was used for the trabecular padding in SV3. Hardly any statistical differences in the cement application forces of human and synthetic vertebrae were found. The measured cement application forces were similar to those measured during the development of the bone cement substitute [22] and hardly any increase of the application force due to the resistance of the trabecular spacing could be detected. The syringe, which was used for the cement application, wasn’t a friction-less application system. Application measurements of empty syringes showed mean plunger pushing forces of 2.64 ± 1.39 N which was less than 7% of the lowest cement application forces measured in this study and thus could be abolished. To create a further training scenario, the available custom made synthetic vertebrae were fractured and cement was applied in a smaller bone surrogate cohort. The measurement results did not show a significant change in cement application force, only slight increase in mean cement spread distance and a slight decrease in circularity. Due to the fact, that a clamping system was used to fix the synthetic vertebrae within the hybrid simulator for minimally invasive spine surgeries, no fracturing of the vertebrae was possible. Furthermore, the fractured vertebrae would lead to a cement leakage and thus a heavy contamination of the system. However, some limitations need to be mentioned. For the vertebroplasty tool insertion measurements, only one needle type, an 11 G diamond tip tool, was used. However, care was taken that only sharp instruments were used for the transpedicular insertions. During a surgery, a surgeon applies an individual amount of force to insert the tool into the pedicle. Due to individual feeding forces and speeds, manual insertion measurements of the tools would be more realistic but hardly comparable. All procedural steps, tool insertion, balloon dilation and cement application, are very individual and variable processes. In our automated measurements, these individual processes were strongly abstracted to obtain comparable and reproducible results. By choosing the experimental parameters, we have attempted the real surgical processes with realistic stresses, feed rates and application forces. During the manufacture of the synthetic vertebrae, the varying ingredients were weighed and mixed manually. Due to this, slight variances in the compositions of the cortical as well as the cancellous bone mixtures occurred. However, as already mentioned above, these variations of the material properties are realistic since they are leading to varying training scenarios for the surgeons.

## Conclusion

In conclusion, our findings demonstrate that a model of synthetic vertebral bodies made of a cortical layer and a cancellous open cell foam from polyurethane resin can provide realistic haptic feedback for vertebral augmentation training. The model has shown adequate mechanical properties especially during transpedicular needle insertion, kyphoplasty balloon tamp dilation and vertebroplasty cement application. The measurements identified the material composition with the most suitable mechanical properties. Commercially available bone models made of polyurethane cannot be recommended for vertebral augmentation training due to unrealistic anatomical structures, unrealistic forces during balloon dilatation and impossibility of cement application. The novel vertebrae model will be used within a hybrid simulator for minimally invasive spine surgeries to educate and train novice surgeons.
